# Cultural adaptation of a community‐based advance serious illness planning decision aid to the Quebec context involving end‐users

**DOI:** 10.1111/hex.13447

**Published:** 2022-02-02

**Authors:** Ariane Plaisance, Yoanna Skrobik, Mathieu Moreau, Felix Pageau, Diane Tapp, Daren K. Heyland

**Affiliations:** ^1^ Faculty of Nursing Laval University Quebec City Quebec Canada; ^2^ Faculty of Medicine McGill University Montreal Quebec Canada; ^3^ Centre Intégré Universitaire de Santé et de Services Sociaux du Nord‐de‐l'Île‐de‐Montréal Montreal Quebec Canada; ^4^ Centre Intégré de Santé et de Services Sociaux de Laval Laval Quebec Canada; ^5^ Department of Family Medicine and Emergency Medicine Faculty of Medicine, University of Montreal Montreal Quebec Canada; ^6^ VITAM Research Center on Sustainable Health, Quebec Integrated University Health and Social Services Center (CIUSSS de la Capitale‐Nationale) Quebec City Quebec Canada; ^7^ Faculty of Medicine University Laval Quebec City Quebec Canada; ^8^ Research Center Institut Universitaire de Cardiologie et de Pneumologie de Québec (IUCPQ) Quebec City Quebec Canada; ^9^ Department of Critical Care Medicine Queen's University Kingston Ontario Canada

**Keywords:** advance care planning, advance serious illness planning, cultural adaptation, decision aid tools, end‐users involvement end‐of‐life care

## Abstract

**Introduction:**

Traditional advance care planning focuses on end‐of‐life planning in the context of a certain or imminent death. It is not tailored for serious illness planning, where the ‘death’ outcome is uncertain. The Plan Well Guide™ (PWG) is a decision aid that empowers lay persons to better understand different types of care and prepares them, and their substitute decision‐makers, to express both their authentic values and informed treatment preferences in anticipation of serious illness. A cultural adaptation was necessary to make the material suitable to the context of Quebec, a French‐speaking Canadian province.

**Methods:**

We engaged lay collaborators and experts in a panel, involving three phases of consultation and data collection. These included an online questionnaire, focused interviews and virtual focus groups that identified elements within the francophone PWG affecting its feasibility, adaptation and integration, as well as items that should be modified.

**Results:**

We engaged 22 collaborators between April and September 2021. The majority (82%) ranked the first translation as good or very good; most (70%) stated that they would recommend the final adaptation. Both lay and expert panel members suggested simplifying the language and framing the tool better within the context of other advance medical planning processes in Quebec. Translation was considered in a cultural context; the challenges identified by the research team or by collaborators were addressed during the focus group. Examples of wording that required discussion include translating ‘getting the medical care that's right for you’ when referring to the PWG's goal. An equivalent expression in the French translation was believed to invoke religious associations. Using the term ‘machines’ to describe life‐sustaining treatments was also deliberated.

**Conclusion:**

Our collaborative iterative adaptation process led to the first French advanced serious illness planning tool. How acceptable and user‐friendly this French adaptation of the PWG is in various Canadian French‐speaking environments requires further study.

**Contribution:**

We organized a focus group inviting both lay collaborators and experts to contribute to the interpretation of the results of the previous phases. This choice allowed us to add more value to our results and to the final PWG in French.

## 1 INTRODUCTION

The global COVID‐19 pandemic means that many individuals will face sudden serious illness. Approximately 5% of COVID‐19‐positive patients will require intensive care unit admission.^1^ In mechanically ventilated patients, 50%–97% do not survive.^2^, ^3^, ^4^, ^5^ Most mechanically ventilated patient deaths are preceded by a decision to withhold or withdraw life‐sustaining technologies. Frequently, family members are called upon by healthcare providers to make life and death decisions on behalf of their loved ones.^6^, ^7^

Serious illness is characterized by uncertainty. When medical decisions are made, no one knows with certainty if the patient will live or die or how survivors will remain affected by long‐term crippling outcomes.^8^, ^9^ In such cases, advance care planning completed under conditions of a certain death or permanent condition may not be valid, resulting in medical error and increased stress by surrogate decision‐makers trying to translate end‐of‐life plans into the serious illness context.^8^, ^9^

When a patient is seriously ill, shared decision‐making between clinicians and patients and/or their family is recommended.^10^ Shared decision‐making is a bilateral exchange between a patient and a clinician. The patient provides insight into their goals, values and preferences, while the clinician outlines the benefits, risks and uncertainties of various treatment options based on their experience and available scientific evidence, and, ideally, framing them within patient preferences.^11^ Clinicians formulate recommendations; they also decide which treatments are most suitable with the patient.^12^ As most seriously ill patients are incapacitated by their condition and thus unable to participate in decision‐making with their clinicians, a substitute decision‐maker represents the patient's values and preferences.^13^ Unfortunately, most substitute decision‐makers are ill‐prepared to face these difficult decisions. This leads to trauma and negative long‐term health consequences.^14^

A common barrier for clinicians to talking to their patients about serious illness planning is their belief that the patient and/or family may not wish to discuss or is unprepared for such a process.^15^ A consultative agency whose mission is to stimulate excellence and the efficient use of resources in the health and social services field in Quebec^16^ conducted a public consultation about goals of care process.^17^ Goals of care process relates to iterative, longitudinal and person‐centred decisions regarding the goals of one's care (from living at all cost to ensuring comfort without prolonging life) that occur between a patient and healthcare providers.^18^, ^19^ Overall results showed that most respondents confirmed their interest in participating in the decisions regarding the intensity of their care. Respondents described past challenges when attempting to ensure that the values and preferences of their loved ones were respected.^17^ Following this consultation, the same agency launched guidelines highlighting the need to prepare lay persons to eventually support a loved one, or themselves, as to how best to decide their preferred intensity of their care in case of serious illness.^19^

Promotion of tools empowering lay persons, their loved ones and surrogate decision‐makers to face future serious illness decisions aims to increase their decisional readiness and facilitate shared decision‐making at the point of care.^8^, ^20^, ^21^ The Plan Well Guide™ (PWG) helps lay persons demystify the different types of care and prepares them (or their substitute decision‐makers) to be able to express their authentic values and informed treatment preferences. By doing so, patients and carers are more decisionally ready in advance of a health crisis.^8^, ^21^ PWG is an evidence‐based tool developed within the framework of the domains and items of the International Patient Decision Aid Standards criteria.^22^ The involvement of end‐users and healthcare professionals across Canada was key in the development of the tool in English.^8^ PWG can be used by lay persons with or without professional assistance. It is currently used and promoted in English‐speaking Canadian provinces. PWG's user data show that the most engaged audience are over 50 years of age with more than a high school education (Daren Heyland [personal communication, May 4, 2021]).

In 2015, the province of Quebec became the first province in Canada to legalize assisted dying.^23^ By positioning voluntary euthanasia as a medical act named Medical Aid in Dying (MAiD), therefore under provincial jurisdiction, Quebec bypassed Canadian criminal law, which, at that time, prohibited ending a person's life.^24^ While Quebec used to be one of the world's most religiously active societies, the quiet revolution in the 1960s led to intense socio‐political and sociocultural changes. In 2019, Quebec's National Assembly voted and adopted the Act respecting the secularism of the State, which enacts ‘laicity’ as a core value overruling certain rights and freedoms. The proposed bill faced stiff opposition in the rest of Canada.^25^ A cultural adaptation was necessary to make the material suitable to the context of Quebec, a French‐speaking province with its own laws and healthcare organization. Adapting knowledge tools to local context is crucial in knowledge translation because it enhances applicability and increases the likelihood of future uptake.^26^, ^27^

## 2 METHODS

### 2.1 Aim

We aimed to culturally adapt PWG to the needs of Francophones in Quebec. We mobilized a panel of experts and a panel of lay collaborators whom we considered as potential users and promoters of the final adaptation of the PWG.

### 2.2 Design

This was a prospective, multiphase, iterative, mixed‐methods study based on the principles of user‐centred design.^28^ and borrowing from the principles of Community‐based Participatory Research.^29^ The research team was composed of a Francophone female (main author) living in the province of Quebec, Canada, trained in community health and qualitative research, and an Anglophone (principal investigator) male living in Alberta, Canada, trained in intensive care medicine and epidemiology.

### 2.3 Setting

This community‐based research was conducted 100% online and over the phone during the COVID‐19 pandemic. The 27 collaborators were in all parts of the province of Quebec, but mainly in Quebec City and Montreal's greater areas.

### 2.4 Collaborators' characteristics

For the expert panel, given the wide scope of advance serious illness planning, we involved experts with diverse complementary backgrounds and expertise such as ethicists, physicians, nurses, social workers, law and community health and healthcare managers living in the province of Quebec, and fluent in read and spoken French.

For lay collaborators, given that we considered the collaborators as potential users of the final adaptation, we involved persons aged over 50 years with at least 13 years of education (1 year of postsecondary studies) in line with the current audience of the original PWG. We justify this choice, given that advance care planning holds greater interest for older individuals.^30^

### 2.5 Sampling and recruitment

We contacted current and former collaborators we knew to be knowledgeable about the topic in this study and requested their participation. We thus used a purposive sampling strategy.^31^

For lay collaborators, we used a convenience sampling strategy.^32^ First, we ran a sponsored Facebook ad (Appendix 1) displayed on the PWG page. We used the platform's settings to ensure that our ad was seen by those fulfilling our inclusion criteria. Then, we invited experts to promote participation of their relatives and friends (word of mouth). Finally, we partnered with the president of a not‐for‐profit organization involved in graduated studies for the elderly (Association des étudiantes et étudiants de l'Université du 3e âge du Québec). We engaged the first 15 individuals whose description fulfilled our inclusion criteria.

### 2.6 Data collection and analysis

**Figure 1:**
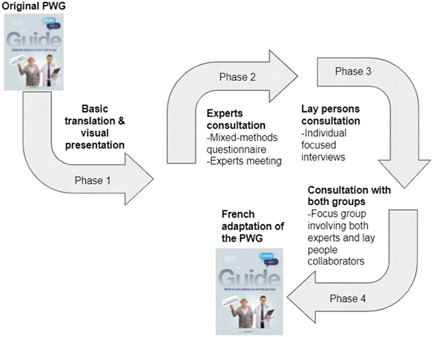
Adaptation process. PWG, Plan Well Guide

We conducted four phases of integrated data collection and analysis (Figure 1). During and after each phase, two team members (A. P. and D. K. H.) met to discuss the results, proposed content changes to the PWG to improve both the original English version and its French adaptation and plan the next steps (10 meetings).

### 2.7 Phase 1: Translation and visual presentation

We translated the PWG and slightly adapted some targeted content. Finally, we added an easily modifiable visual template copying the original PWG.

### 2.8 Phase 2: Experts consultation

#### 2.8.1 Mixed‐methods questionnaire

We developed an online mixed‐method questionnaire for experts based on previous work^33^, ^34^, ^35^, ^36^ (Appendix 2). The questionnaire intended to assess experts' views on the usability, acceptability and comprehensibility of the plain translation of PWG, as well as their insight on linguistic and anthropological meaning associated with direct translation. Respondents were requested to answer the questionnaire from the perspective of the current audience of the original PWG (over 50 with at least 13 years of education). Respondents were invited to look at the final graphics in the original English guide. We used Qualtrics (www.qualtrics.com) to administer the questionnaire from 7 May to 21 May 2021.

### 2.9 Experts meeting

Following the questionnaire analysis, we organized a meeting with the expert panel with  the aim of discussing the results and reflect on specific meanings that the translation may have influenced. This 2‐h Zoom (31 May 2021) meeting was conducted in French.

### 2.10 Readability assessment

We assessed the readability of the final adaptation using Scolarius online software (www.scolarius.com), where readability scores range from 50 to 190+. The score reflects the educational level required for comprehension. For instance, a score of 50 reflects comprehension by readers with elementary school education, and a score of 190+ reflects that the targeted population is an expert in the field.

### 2.11 Phase 3: Lay persons' consultation

We developed an individual‐focused interview grid similar to the questionnaire used with experts. The interviews intended to assess lay collaborators' views on the usability, acceptability and understandability of the plain translation of PWG. Respondents were also invited to look at the final graphics in the original English guide. Zoom interface or telephone interviews were held between 16 June and 12 July (*n* = 9 and *n* = 6, respectively) based on collaborator preference.

Following the two consultation rounds, we modified the French content and hired a professional graphic designer to create a working version of the French adaptation of PWG.

### 2.12 Phase 4: Focus group mixing lay persons and experts

We invited interested collaborators in both panels to continue their involvement through a focus group.^37^ The focus group aimed to introduce and discuss some results of the study, to collect their comments and suggestions of improvement of the working version of the French adaptation of PWG including a ‘Frequently Asked Question’ (FAQ) complement that was developed during the adaptation process. This 2‐h Zoom (13 September 2021) meeting was held in French.

Following the focus group, we asked the graphic design professional to create the final version of the French adaptation of PWG and we updated the FAQ complement based on the results of the focus group.

## 3 RESULTS

The study was conducted from April to September 2021. The 27 collaborators were located mainly in Quebec City and Montreal greater areas (Table 1).

**Table 1:** Sociodemographic information of collaborators

Expert panel^a^	*N* = 12
Expertise	
Nurses	4
Ethicists	3
Palliative care specialist	3
Physician specialized in intensive care	2
Physician specialized in palliative care	1
Geriatrician	1
Social anthropologist	1
Primary care physician	1
Social worker	1
Nutritionist	1
University professor in law	1
University professor in community health	1
Decision‐maker	1
Health‐care administrator	1
Gender	
Female, *n* (%)	9 (75)

Lay persons' panel	*N* = 15
Age, mean (range)	68.3 (27)
Gender	
Female, *n* (%)	11 (73.3)
Country of birth	
Canada, *n* (%)	14 (93.3)
Education	
More than 18 years (bachelor degree), *n* (%)	10 (66.7)
20 years (master degree), *n* (%)	4 (26.7)
More than 24 years (doctoral degree), *n* (%)	1 (6.7)
Background	
Healthcare technicians and professionals, *n* (%)	5 (33.3)
Teachers, *n* (%)	3 (20.0)
Lawyers, *n* (%)	2 (13.3)
Other, *n* (%)	5 (33.3)

^a^ The total number of expertise is higher than the number of experts because a few experts had expertise in many areas.

### 3.1 Phase 1: Translation and visual presentation

During the translation process, we adapted some targeted content. Table 2 introduces the changes that were agreed upon with their rationale.

**Table 2:** Changes during the translation process and rationale

English version	Decision	Rationale
Use of the wording *doctors* as interlocutors throughout the documents	In statements about values and preferences elucidation and discussion, the wording *équipe soignante* (English: care provider team) would be used. In statements about level of care decisions conducted within the healthcare system, we agreed to use the wording ‘médecins’ (English: Physicians)	We wished to emphasize the diversity of the many healthcare professionals competent in supporting and eliciting values and preferences and to discuss Advanced Serious Illness Planning as supported by recent literature.^38^, ^39^, ^40^ At the time of this study, the final decisions and form signing were legally reserved to physicians in Quebec. Since then, nurse practitioners have acquired professional recognition to conduct this process, including form signing^41^
Use of the wording *Cardiorespiratory resuscitation*	Use of the wording: *Tentative de réanimation cardiorespiratoire* (English: Attempt cardiorespiratory resuscitation)	To highlight the inherent reality that any resuscitative measure is an effort with no guarantee of successful results
Cardiopulmonary resuscitation is generally a part of intensive care, but can be provided in other parts of the hospital as well	We altered this to: *La réanimation cardiorespiratoire est tentée par défaut dans tous les milieux de soins si le cœur d'une personne s'arrête* (English: Cardiopulmonary resuscitation is attempted by default in all healthcare settings if a person's heart stops)	To explicitly state that the overall default treatment, given its potential for saving lives and the time‐sensitive nature of cardiopulmonary resuscitation (CPR), in the event of cardiac arrest, contrasts with all other medical interventions for which consent is required. For CPR, a medical order is a prerequisite to not attempt it^42^

### 3.2 Phase 2: Experts' consultation

#### 3.2.1 Questionnaire

Eight (8) experts (ages 34–64 years, 6 females) replied to the questionnaire (75% response rate). Most experts (75%) thought that the PWG was either good or very good; half (50%) indicated that they would probably or definitely recommend the proposed final version to others (Table 4).

##### 3.2.1.1 Analysis of the open‐ended questions

Three main issues emerged from the analysis of the open questions. The need to simplify the language and to reduce the length of the text were considered an important goal, and second, anchoring the tool better in the context of other advance planning processes in Quebec to avoid creating more confusion in an already complex environment (e.g., advance medical directives, goals of care forms, living will, available through notarial and provincial networks). Even though they did not express it, some collaborators seemed unclear about the differences between the concept of Advance Serious Illness Planning and the concept of traditional Advance Care Planning. Through these observations, the need to further clarify the concept of Advance Serious Illness Planning versus Advance Care Planning emerged as the third issue.

##### 3.2.1.2 Insight on translation challenges

Individual experts' insight was sought to translate some headlines of the original version of PWG such as the headlines *Getting the medical care that's right for you* and *In order to keep you ticking, we need to know what makes you tick*, chosen in English for their appeal, translated poorly and seemed culturally maladapted.

Apart from agreeing that the title *PWG* should be translated into French, no consensus was arrived at on the other translation challenges. As a result, we submitted the translation challenges to the expert panel.

#### 3.2.2 Experts' meeting

Nine experts attended; nine translation challenges were presented. The translation challenges, agreements and rationales are presented in Table 3.

**Table 3:** Translation challenges discussed with experts, agreements and rationales

Original English version	First translation	Challenge/issue submitted to experts	Agreement and rationale
Getting the medical care that's right for you	*Obtenir les soins qui correspondent à vos valeurs et préférences* (English: Getting the care that matches your values and preferences)	Not the direct translation of *Getting the medical care that's right for you* need to find a translation closer to the English wording without adding complexity	**Obtenir les soins qui sont bons pour vous** (English: Getting the care that's right for you) The translation is closer to the English version
Plan Well	Was not translated	Needed to find an appealing title	**Planifiez bien** (First‐person plural imperative form) (English: Plan Well) We thought using the imperative would encourage involvement and action for the reader
To live and die well, you need to Plan Well	*Pour bien vivre et bien mourir, il faut bien planifier* (English: To live and die well, you need to Plan Well)	One expert suggested: *Pour bien vivre jusqu'à la fin, il faut bien planifier* in the online consultation. We discussed this suggestion. (English: To live well until the end, you have to plan well)	**Pour bien vivre et bien mourir, il faut bien planifier**. (English: To live and die well, you need to Plan Well) We did not want to remove the word *die* as suggested by the expert. The expert group wished to avoid using the ‘until the end' euphemism
To keep you ticking, we need to know what makes you tick	*Pour pouvoir maintenir votre qualité de vie, nous avons besoin de savoir comment vous définissez la qualité de vie* (English: To be able to maintain your quality of life, we need to know how you define quality of life)	Needed to find an appealing slogan closer to the English version	**Afin de vous garder actif, nous avons besoin de savoir ce qui vous active** (English: In order to keep you active, we need to know what activates you) It uses the same wording in both parts as in the English version
Try for a bit	Direct translation: *Essayez un peu* (English: Try for a bit)	One expert suggested in the online consultation to rephrase this without using direct translation	**Juste un peu** (English: Just a little bit) Sounds better
Easy does it	Direct translation: Facile, ça le fait (English: Easy does it)	One expert suggested in the online consultation to rephrase this without using direct translation	**Pas d'acharnement** (English: No heroic measures) This is a very common statement used in vernacular and legal language in Quebec to describe the wish to avoid excessively invasive measures [43]
Substitute decision‐maker	*Mandataire en cas d'inaptitude* (English: Mandator in case of incapacity)	*Mandataire en cas d'inaptitude* was not the correct translation for *Substitute Decision‐Maker* as it was pointed out by some experts in the online questionnaire	**Représentant** (English: representative) We wished to stay inclusive of official Substitute Decision‐Maker and unofficial representative (e.g. family members who can be called to play a decisional role without being the official Substitute Decision‐Maker
Life‐sustaining treatments	Interventions de maintien des fonctions vitales (English: Life‐support interventions)	One expert suggested in the online consultation to simplify the wording used because they though lay persons would not understand	**Interventions visant la prolongation de la vie** (English: Life‐prolonging interventions) We chose this wording because it is the same as that used on the official goals of care form in Quebec [19]We added a footnote stating: *des interventions telles la réanimation cardiorespiratoire, l'intubation et la dialyse* (English: interventions such as cardiopulmonary resuscitation, intubation and dialysis)
Outcomes	*Les suites de…* (English: The suites of…)	Experts made several online suggestions in the online consultation: ce qui arrive après X, le résultat de X mène/donne Y, après l'évolution anticipée de l'état de santé, conséquences (English: what happens after X, the result of X leads/gives Y, after the anticipated evolution of health status, health, consequences or outcomes	**Ce qui est attendu après** (In English: What is expected following) This wording differs from the suggestion, and was determined through subsequent discussion

### 3.3 Phase 3: Lay persons' consultation

Most (80%) lay persons thought that the first translation of the PWG was either good or very good. The same proportion (80%) indicated that they would probably or definitely recommend the proposed final version to others (Table 4).

**Table 4:** Closed‐ended questions for both groups and overall

**Questions**	Experts (*N* = 8)	Lay persons (*N* = 15)	Total (*N* = 23)
How clear is the language used?			
Very unclear/unclear, *n* (%)	0 (0)	1 (6.7)	1 (4.3)
Neither clear nor unclear, *n* (%)	1 (12.5)	3 (20)	4 (17.4)
Clear/very clear, *n* (%)	7 (87.5)	11 (73.3)	18 (78.2)
How likely would you be to recommend the final version of PWG to someone?			
Definitely/probably would not recommend, *n* (%)	0 (0)	1 (6.6)	1 (4.3)
Might recommend, *n* (%)	4 (50)	2 (13.3)	6 (26.0)
Probably/definitely would recommend, *n* (%)	4 (50)	12 (80)	16 (69.6)
Overall, how would you rate the guide?			
Very poor/poor, *n* (%)	0 (0)	0	0 (0)
Fair, *n* (%)	2 (25.0)	3 (20.0)	5 (21.7)
Good/very good, *n* (%)	6 (75.0)	12 (80.0)	18 (78.3)

#### 3.3.1 Analysis of the open‐ended questions

Four main issues emerged from the interviews with lay persons. First, there was a need to simplify the language. Second, there was an expressed need to clarify the role of general practitioners in the process of Advance Serious Illness Planning. Four persons indicated not having access to a general practitioner and two thought that their general practitioner would not be the best person with whom to discuss such matters. Finally, they also requested that PWG be better situated in the context of other advance medical planning processes in Quebec (e.g., goals of care, advance medical directives).

Most lay persons indicated that they learned a lot during their participation in the research project, with six highlighting that, before their participation, they never heard about the possibility to participate in decisions regarding the intensity of their care in case of serious illness. As observed in the expert panel, at least two persons seemed to struggle with the differences between the concept of Advance Serious Illness Planning and the concept of Advance Care Planning. Thus, the need to further clarify the concept of Advance Serious Illness Planning versus Advance Care Planning emerged as the fourth issue.

#### 3.3.2 Results of the closed‐ended questions

Table 4 presents the results of the closed‐ended questions for both groups and overall.

### 3.4 FAQ complement and graphic design

Aiming to address the needs we could not include in the PWG such as the optional role of general practitioners and the remaining confusion regarding the concept of Advanced Serious Illness Planning, we developed a FAQs complement. It is based on the FAQ page previously available at www.planwellguide.com and adapted to the needs that we identified and to Quebec laws and regulations.

### 3.5 Phase 4: Focus group

Six panellists (four experts, two lay persons) participated. Some concerns and discomfort were specific to lay panellists. One example was the translation of ‘right for you’ into ‘bon pour vous’ (English: good for you) when referring to the goal of PWG (to help people get the medical care that's right for them), which was first debated during the expert meeting. A lay person raised how problematic this phrasing seemed. This individual described discomfort at the wording ‘bon’ (English: good) because of its religious roots (good and evil). Other collaborators agreed emphatically with this lay person's opinion. One expert then suggested using the wording *appropriés pour vous* (English: appropriate for you), which defines care as medically appropriate and coherent with a patient's values and preferences according to the Medical college of Quebec.^43^ This was deemed acceptable.

Two lay collaborators and one expert disliked the *machines* wording (English: machines) throughout the document to describe intensive care technologies used to replace a failing organ and suggested rather using the wording *appareils* (English: devices). They thought that the wording *machine* relates more to a garage fixing cars than to a hospital. Two experts with current clinical practices described using the wording *machine* daily with their patients, with the conviction that this term was more comprehensible to lay persons. They disagreed with the *appareils* (English: devices) wording suggestion because they thought that it trivialized the invasive technical interventions and thus did not reflect the reality of intensive care units. We compromised by first stating *machines* in brackets and adding a footnote explaining the word's intended meaning (Machines used in intensive care are medical devices that temporarily replace a failing organ (e.g.,: ventilator for the lungs, dialysis for the kidneys, etc.).

### 3.6 Readability assessment

The final adaptation received a score of 142, reflecting that the document is fully comprehensible for someone who completed 13 years of education, which is slightly higher than the level of education of the current audience of the English version of PWG (12 years of education).

## 4 DISCUSSION

This iterative adaptation process involving experts and lay persons whom we considered potential users and promoters of the final adaptation of the PWG led to the development of the first Advance Serious Illness Planning tool available in French. The final PWG takes into account the generally frequent lack of knowledge regarding cardiopulmonary resuscitation such as its low efficacy to bring patients back to life and in the same state as they were before dying^44^ and the requirement to get a medical order not to attempt it as opposed to other interventions.^45^ Moreover, it is sensitive to the discomfort of most of Quebec population with wording relating to religion or invoking religious associations. We identified users' needs and collected suggestions during three phases of consultation and successfully addressed most.

### 4.1 Trouble understanding Advance Serious Illness Planning

We observed through the questions and suggestions of end‐users from both groups that some collaborators did not understand the concept of Advance Serious Illness Planning even after reading the first pages of PWG that are dedicated to explaining this new concept. The complex and new notion of Advance Serious Illness Planning, which requires a clear perception that death is a potential, rather than certain, outcome within a hypothetical future episode of care, was difficult to conceptualize for some collaborators. This result is not surprising since most of the plans or medical directives that people have been in contact with are framed around conditions of a certain death.^21^ Such trouble understanding of the concept of Advance Serious Illness Planning was also observed during the development of the English version.^8^ To address the remaining incomprehension while avoiding creating more complexity in the main document, we developed an FAQ complement where the iterative nature of Advance Serious Illness Planning is clearly and briefly explained.

### 4.2 Acceptability of PWG but need to situate in the current medical decision‐making landscape in Quebec

From the beginning of the process, both groups of stakeholders had a favourable opinion towards the PWG and indicated that they would recommend the final adaptation to others. However, they asked us to better situate the tool in the already complex landscape of advance medical planning processes in Quebec. Indeed, in the last decade, the environment of advance medical planning processes has become increasingly more complex in this province. First, in 2015, the *Act respecting end‐of‐life care* led to the creation of a national register of advanced medical directives. Advanced medical directives are put into place if the person is no longer capable of giving consent to care, carried out without professional guidance and overrule the consent of the person's representative.^23^ Then, in 2016, a standardized goals of care form aiming to replace a myriad of local and regional forms was implemented in the province.^19^ As opposed to advanced medical directives that are carried out without professional guidance, goals of care forms are completed in an institutionalized setting and involve patients and/or substitute decision‐makers, physicians and sometimes other healthcare professionals and providers.^18^, ^38^, ^46^ Despite this complexity, and even though provincial reports have highlighted the need to improve the knowledge of the population towards advance medical planning processes,^17^ little action has been taken. To avoid creating more complexity in the main document, we added precision regarding the embedment of the PWG into the current medical decision‐making landscape in Quebec in the FAQ complement.

### 4.3 Role of the general practitioner in Advance Serious Illness Planning

Four persons indicated not having access to a general practitioner and two thought that their general practitioner would not be the best person with whom to discuss such matters. Even though the current government committed in 2018 to provide all Quebecers with a general practitioner by the end of its mandate, this promise has yet to become a reality. In 2018, 400,000 patients were on a waiting list for a physician. This number has since doubled to over 800,000,^47^ making it difficult, if not impossible, to realistically pair primary care physicians with patients in a goals‐of‐care process. We therefore specified in the FAQ complement that no access to a general practitioner is not problematic since their general practitioner is not likely to be the one treating them during an episode of serious illness. Instead, another physician working in the emergency room or intensive care unit would be most interested in the content or information within their completed PWG.

Our translation challenges were resolved thanks to the sincere involvement of collaborators with diverse expertise and backgrounds who openly shared their thoughts and worked together as peers. Other studies involving several groups of stakeholders highlighted the importance of creating a receptive context by integrating each of the stakeholder groups in an ‘egalitarian spirit’.^48^ Lay persons also seemed to have appreciated their involvement.

We believe that the acceptability of advance serious illness planning reflected in the lay person panel's support of how ‘good’ the translated tool is and how they learned from it, the challenge of accessing primary care in Quebec and the need to educate and empower patients and families support the implementation of the PWG in this province.

### 4.4 Strengths

Our results could support researchers aiming to adapt decision aids culturally. Currently, no guidelines guide researchers and developers in adapting existing decision aids to new languages and contexts.^49^ As a result, some have called for more transparent and complete reporting.^28^, ^50^ We intentionally introduced our methodology and results using thick description^51^ to fill this knowledge gap, as few publications detail the steps required to create or to adapt decision aids.

We mobilized experts and lay persons whom we considered as potential users and promoters of the final adaptation of the PWG. We organized a focus group inviting both lay collaborators and experts. As highlighted in theoretical orientations of patient and public involvement, for experiential knowledge to gain its full value, a space must exist where experts and lay persons meet on equal grounds.^52^ This choice allowed us to add more value to our results and to the final PWG in French.

### 4.5 Limitation

Due to the global COVID‐19 pandemic, we conducted this study 100% virtually and our project did not allow for in‐person interaction. Meeting collaborators solely online limited our capacity to have meaningful interaction with them and to interpret their nonverbal language. In human communication, nonverbal signs are as critical as actual spoken words.^53^ These limits have been documented in research about the pros and cons of online meetings during the COVID‐19 pandemic.^54^

Our lay collaborators were more educated than the persons we intended to collaborate with, as all had attended university and held bachelor's, master's and doctoral degrees (over 18 years of education). Partnering with the president of a not‐for‐profit organization involved in graduate studies for the elderly was partially linked to this situation. Therefore, we could not capture and address the difficulties and concerns of our targeted audience, leading to limits in the generalizability of the final adaptation. The software analysis suggested that the final adaptation is fully comprehensible for someone who completed 13 years of education, as was the case for the English version of the PWG. The inherent limitations of such software, such as they are not evaluating functional literacy or true comprehension of content or meaning, limit this aspect of our analysis and will require further evaluation in subsequent studies.

## 5 CONCLUSION

All Canadians deserve access to clear and useful information about the different options for care in case of serious illness. The PWG™ helps lay individuals demystify different types of care and prepares them (and their substitute decision‐makers) to express their authentic values and informed treatment preferences when they develop serious illness. Our collaborative iterative adaptation process involving end‐users led to the development of the first Advanced Serious Illness Planning tool available in French. To address the information needs that we could not include in the PWG, such as the optional role of general practitioners and the remaining confusion regarding the concept of Advanced Serious Illness Planning, we also developed a ‘Frequently Asked Question’ complement that is Quebec‐specific. However, the PWG in French remains transposable to other French‐speaking communities in other Canadian provinces. Future research includes assessment of the acceptability and usability of the French adaptation of the PWG in various French‐speaking clinical and professional environments in various Canadian provinces before a more comprehensive evaluation of its effectiveness.

## CONFLICT OF INTERESTS

The authors declare no conflicts of interest.

## ETHICS STATEMENT

The project was approved by Queen's University Health Sciences and Affiliated Teaching Hospitals Ethics Board (#6013436).

## AUTHOR CONTRIBUTIONS

Ariane Plaisance and Daren K. Heyland were involved in the *conception, design, data collection, interpretation, drafting and review of the manuscript*. Yoanna Skrobik, Mathieu Moreau, Félix Pageau and Diane Tapp were involved in *Data collection, manuscript drafting and review*.

## Data Availability

The data sets during and/or analysed during the current study are available from the corresponding author on reasonable request.

## APPENDIX 1 FACEBOOK ADS

RESEARCH PROJECT. A research team from Queens's university (Ontario) wants to adapt an innovative guide on serious illness planning to the needs of a francophone audience. If you are over 50, capable of writing and reading French and if you completed at least 1 year of postsecondary studies, you could contribute to improve the respect of values and preferences of persons facing life‐threatening conditions like COVID‐19 pneumonia! Your involvement would take around 90 min of your time and will be 100% online or over the phone. Please contact the principal investigator at dkh2@queensu.ca.

## APPENDIX 2 EXPERTS QUESTIONNAIRE

Part I (sociodemographic)

This set of questions is intended to provide a sociodemographic picture of experts

1. In which year were you born?
2. Which gender do you identify with?
3. What is your profession?

Part II (overall assessment)

This set of questions will ask about the entire guide so try to keep the whole experience in mind as you answer them. Please answer according to the target audience (persons aged 50 and over who completed at least 1 year of postsecondary studies).

4. How clear is the language used?
  **Table:**
  **Very unclear 1**	**Unclear 2**	**Neither clear nor unclear 3**	**Clear 4**	**Very clear 5**
5. Are there specific things you would like to report as being unclear?
  *(medium paragraph)*
6. How do you like the images used in the Plan Well Guide? You can take a look at the final appearance by consulting the guide in English.
  *(medium paragraph)*
7. What things should be added to the Plan Well Guide?
  *(medium paragraph)*
8. What things should be removed from the Plan Well Guide?
  *(medium paragraph)*
9. Overall, how difficult or easy do you think it would be for the target audience to use the Plan Well Guide?
  **Table:**
  **Very difficult** **1**	**Difficult** **2**	**Neither difficult or easy** **3**	**Easy** **4**	**Very easy** **5**
10. Is there a specific part that you think would be particularly difficult to make sense of for the target audience?
  *(medium paragraph)*

11. a) How likely would you be to recommend the final version of Plan Well Guide to someone?
  **Table:**
  **Definitely would not recommend** **1**	**Probably would not recommend** **2**	**Might recommend** **3**	**Probably would recommend** **4**	**Definitely would recommend** **5**
b) Why?
  *(medium paragraph)*

12. Overall, how would you rate the guide?
  **Table:**
  **Very poor** **1**	**Poor** **2**	**Fair** **3**	**Good** **4**	**Very good** **5**
13. Do you have any other thoughts or comments about the Plan Well Guide that you want to tell us about? Any suggestions for improvement?
  *(long paragraph)*
  Part III (translation challenges)
14. Shall we translate the title of the guide (Plan Well Guide) or keep it in English?
  *(closed‐ended question)*

a)  Yes, translate it
b)  No, it's fine in English

*Conditional link: If they replied (a), go to question 15. If they replied (b) go to question 17*

15. Is the headline ‘Bien planifier’ as a translation of ‘Plan Well’ appropriate?
  *(closed‐ended question)*

a)  Yes
b)  No

*Conditional link: If they replied (a), go to question 17. If they replied (b), go to question 16*.

16. Can you help us find a catchy headline?
  (short paragraph)
17. Is the translation of the headline ‘In order to keep you ticking, we need to know what makes you tick?’ to ‘To be able to maintain your quality of life, we need to know how you define quality of life?’ appropriate?
  *(closed‐ended question)*

a)  Yes
b)  No

*Conditional link: If they replied (a), go to question 19. If they replied (b), go to question 18*.

18. Can you help us find a better translation?
  *(short paragraph)*
19. Is the translation of ‘To live and die well, you need to Plan Well’ into ‘To live and die well, you need to plan well’ appropriate to you?
  *(closed‐ended question)*

a)  Yes
b)  No

*Conditional link: If they replied (a), go to question 21. If they replied (b), go to question 20*.

20. Can you help us find a catchy headline?
  *(short paragraph)*
21. In French, there is no perfect translation of the English word ‘outcomes’. In the description of the three types of medical care (intensive care, medical care, comfort care), we translated ‘outcomes’ by ‘les suites de’. What do you think of this translation?
  *(short paragraph)*
22. Do you have any other thoughts or comments about the Plan Well Guide that you want to tell us about? Any suggestions for improvement?
  *(long paragraph)*

*Thank you for taking the time to complete this assessment and answer our questions about the Plan Well Guide*
